# Secretion of Mutant DNA and mRNA by the Exosomes of Breast Cancer Cells

**DOI:** 10.3390/molecules26092499

**Published:** 2021-04-25

**Authors:** Olga E. Andreeva, Yuri Y. Shchegolev, Alexander M. Scherbakov, Ekaterina I. Mikhaevich, Danila V. Sorokin, Margarita V. Gudkova, Irina V. Bure, Ekaterina B. Kuznetsova, Dmitry S. Mikhaylenko, Marina V. Nemtsova, Dmitry V. Bagrov, Mikhail A. Krasil’nikov

**Affiliations:** 1 Department of Experimental Tumor Biology, Institute of Carcinogenesis, N.N. Blokhin National Medical Research Center of Oncology of the Ministry of Health of Russia, 115522 Moscow, Russia; tilberta@gmail.com (O.E.A.); yurashhegolev@gmail.com (Y.Y.S.); k.mihaevich@gmail.com (E.I.M.); dsorokin2018@gmail.com (D.V.S.); gudkova@ronc.ru (M.V.G.); krasilnikovm1@yandex.ru (M.A.K.); 2 Laboratory of Medical Genetics, Institute of Molecular Medicine, I.M. Sechenov First Moscow State Medical University (Sechenov University), 119991 Moscow, Russia; bureira@mail.ru (I.V.B.); kuznetsova.k@bk.ru (E.B.K.); dimserg@mail.ru (D.S.M.); nemtsova_m_v@mail.ru (M.V.N.); 3 Department of Bioengineering, Faculty of Biology, Lomonosov Moscow State University, 119234 Moscow, Russia; bagrov@mail.bio.msu.ru

**Keywords:** exosomes, breast cancer cells, DNA transferring, cancer, signalling

## Abstract

Exosomes are the small vesicles that are secreted by different types of normal and tumour cells and can incorporate and transfer their cargo to the recipient cells. The main goal of the present work was to study the tumour exosomes’ ability to accumulate the parent mutant DNA or RNA transcripts with their following transfer to the surrounding cells. The experiments were performed on the MCF7 breast cancer cells that are characterized by the unique coding mutation in the *PIK3CA* gene. Using two independent methods, Sanger sequencing and allele-specific real-time PCR, we revealed the presence of the fragments of the mutant DNA and RNA transcripts in the exosomes secreted by the MCF7 cells. Furthermore, we demonstrated the MCF7 exosomes’ ability to incorporate into the heterologous MDA-MB-231 breast cancer cells supporting the possible transferring of the exosomal cargo into the recipient cells. Sanger sequencing of the DNA from MDA-MB-231 cells (originally bearing a wild type of *PIK3CA*) treated with MCF7 exosomes showed no detectable amount of mutant DNA or RNA; however, using allele-specific real-time PCR, we revealed a minor signal from amplification of a mutant allele, showing a slight increase of mutant DNA in the exosome-treated MDA-MB-231 cells. The results demonstrate the exosome-mediated secretion of the fragments of mutant DNA and mRNA by the cancer cells and the exosomes’ ability to transfer their cargo into the heterologous cells.

## 1. Introduction

Exosomes are among the most popular and perspective objects of recent molecular oncology studies. Exosomes represent the 30–100 nm-sized microvesicles that are generated in the cells, released into the extracellular space, and carry a wide spectrum of bioactive molecules. The most intriguing property of exosomes represents their ability to incorporate into the recipient cells resulting in the cascade of both genomic and nongenomic changes. Among the exosomal cargo, proteins preserving their biological activity after transferring such as ABC transporters, receptor tyrosine kinases, growth factors, etc., and small stable RNAs, primarily microRNAs were of great interest for research. Basing on the different experimental models and clinical data the ability of exosome-transferred molecules to modulate the main properties of the recipient cells that are growth and survival, drug resistance was found to occur [1,2,3,4,5]. 

In addition, the exosomes can transfer the fragments of the parent DNA or mRNA, but their role in the regulation of the recipient cells is unclear and requires further investigation. Concerning cancer cells, certain observations demonstrate the presence of the mutant tumour DNA in the native exosomes [6,7,8,9]; however, the efficiency of mutant DNA accumulation and the possible presence of the mutant RNA transcripts in the exosomes are open questions—as well as the possible involvement of the DNA-enriched exosomes in the gene exchange between the cells. 

Here, the analysis of MCF7 breast cancer cells revealed the presence of the DNA and RNA transcripts with the native *PIK3CA* mutation in the secreted exosomes. We demonstrated the effect of MCF7 exosomes’ incorporation into the heterologous recipient cells supporting the possible transferring of the exosomal cargo into the recipient cells.

## 2. Results

**Figure 1:**
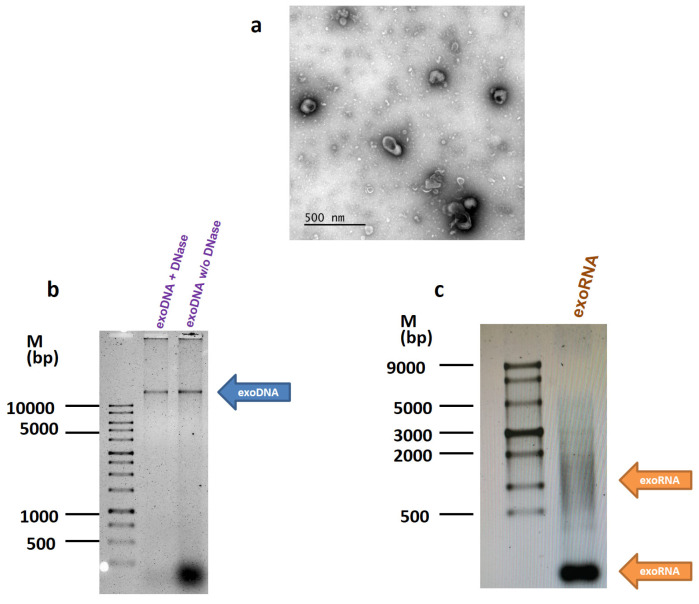
Detection of DNA and RNA in the exosomes. (**a**) The transmission electron microscopy of the exosomes. Exosomes were prepared from the MCF7 conditioned medium by the differential ultracentrifugation, and imaged as described in Methods. (**b**,**c**) DNA and RNA were isolated from the MCF7 exosomes and separated on 0.8% and 1.2% agarose gel, respectively.

The experiments were performed on the MCF7 breast cancer cells characterized by the unique coding mutation in the *PIK3CA* gene. Exosomes were prepared from the MCF7 conditioned medium by the differential ultracentrifugation, and exosome imaging was carried out by transmission electron microscope as described in Methods (Figure 1a). The exosome functional studies were performed as recommended by ISEV in [10] and fully described in [11]. The quality of purified DNA and RNA samples was controlled by separation on the 0.8% and 1.2% agarose gel, respectively (Figure 1b,c). Most DNA was found inside the exosomes and did not degrade after treatment with DNase. 

**Figure 2:**
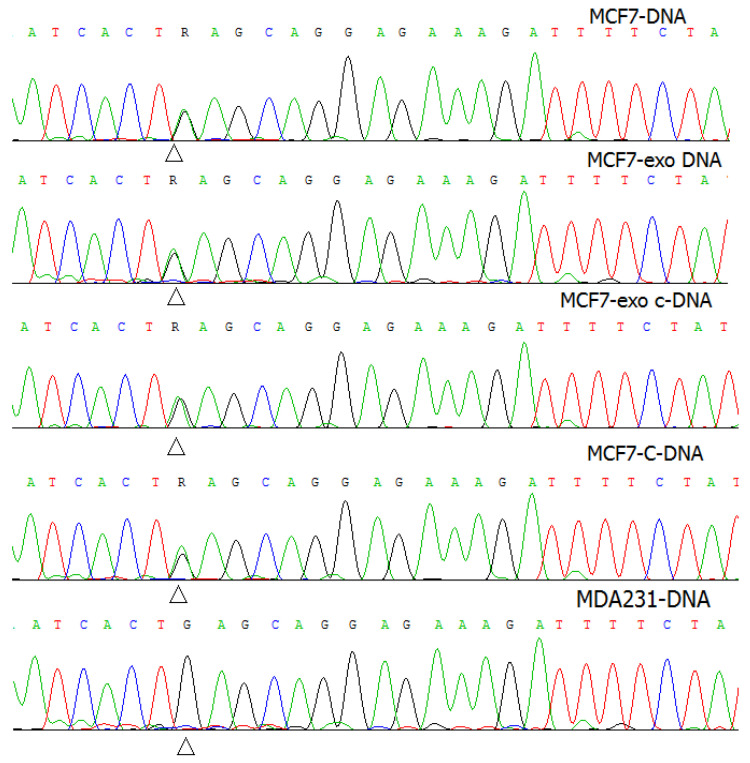
Mutation validation by Sanger sequence analysis. The sequence electrophoregrams of the MCF7-DNA, exosomal DNA from MCF7, cDNA from MCF7, and exosomal cDNA from MCF7 contained the *PIK3CA* mutation c.1633G > A (p.E545K) (R), compared to the MDA-231 DNA.

Sanger sequencing of exosomal DNA and RNA revealed the *PIK3CA* mutation E545K (c.1633G > A) in both DNA and RNA samples (Figure 2). 

**Figure 3:**
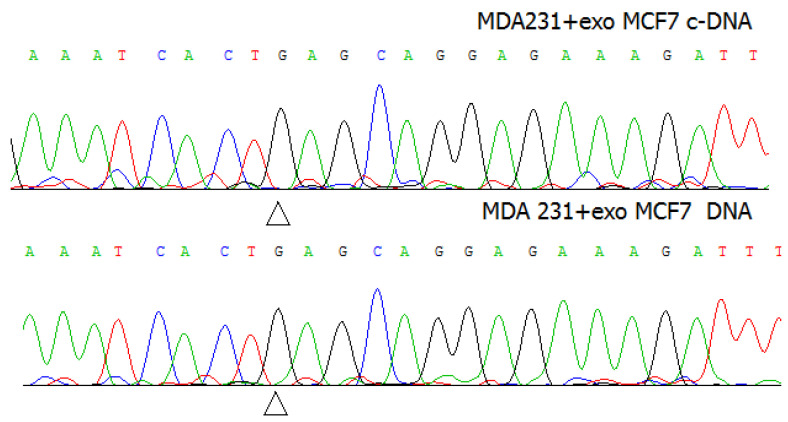
The sequence electrophoregrams of DNA and c-DNA of MDA-MB-231 cells after treatment with the exosomes of MCF7 cells.

Are the exosomes capable to transfer the mutant nucleic acids to the recipient cells? To answer this question, the subsequent experiments were performed using MDA-MB-231 breast cancer cells characterized by the wild type of the *PIK3CA* gene. The MDA-MB-231 cells were treated with the exosomes of MCF7 cells, and cytoplasmic DNA or RNA of MDA-MB-231 cells was isolated and sequenced. There was no detectable amount of mutant DNA or RNA found in the treated MDA-MB-231 cells (Figure 3). 

Furthermore, the *PIK3CA* mutation was detected by an independent method—allele-specific real-time PCR. The threshold cycle (Ct value) was calculated as the cycle when the fluorescence of the sample exceeded a threshold level corresponding to 10 standard deviations from the mean of the baseline fluorescence. We provided three technical replications of the reaction per sample and calculated average Ct, standard deviation, and indexes associated with them according to the Guide for TaqMan^®^ Mutation Detection Assay (Thermo Fisher Scientific, Waltham, MA, USA). The average Ct for all reactions was determined, and the delta Ct of the mutant and reference (normal) alleles was calculated. Then, the cut-off for delta Ct was determined as the total average Ct of the negative control minus three standard deviations. As shown, the delta Ct of MDA-MB-231 cells treated with MCF7 exosomes was less than the cut-off delta Ct of the negative control (MDA-MB-231 cells without *PIK3CA* mutation) in absolute values but did not differ significantly revealing the slight tendency to accumulation of mutant DNA in the exosome-treated cells. Probably, the amount of mutant DNA in the treated cells was under the threshold of analytical sensitivity of the used real-time PCR protocol in this case (Table 1).

**Figure 4:**
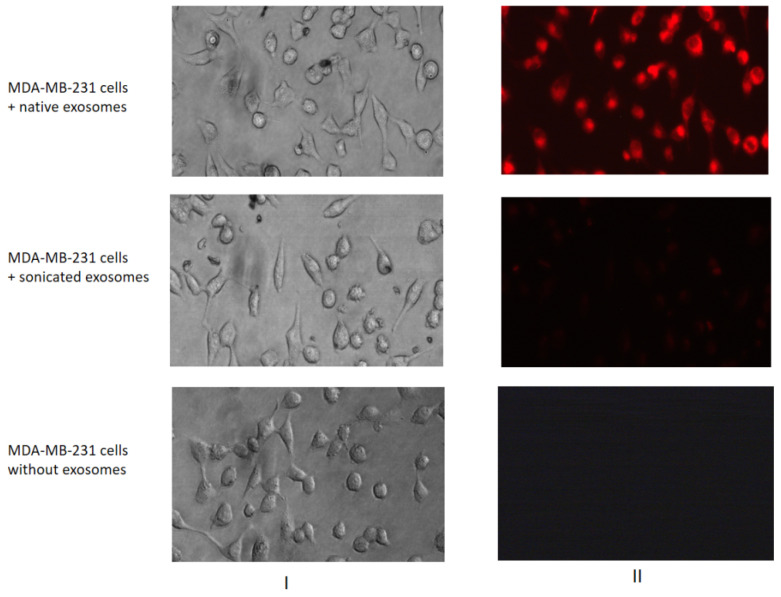
Transferring of fluorescent-labelled compounds by exosomes. MDA-MB-231 cells were cultured without labelled exosomes or with the native or sonicated MCF7 exosomes stained by a fluorescent dye. The efficiency of dyeing exosome incorporation was checked with a fluorescent microscope Nikon Eclipse Ti-E. The images of light (I) and fluorescent (II) microscopy are presented.

To demonstrate the possibility of MCF7 exosomes to incorporate into the MDA-MB-231 cells, the vesicles were labelled by fluorescent dye CellTracker™ Red CMPTX Dye (Thermo Fisher Scientific, Waltham, MA, USA). Then thoroughly stained MCF7 exosomes were washed in PBS twice by the ultracentrifugation 100 000× *g*, and precipitates were dissolved in PBS. One portion of the exosomes was destructed by sonication (used as a negative control), and native and sonicated exosomes were incubated with MDA-MB-231 cells for 1 h. As shown, the MCF7 exosomes were able to accumulate the fluorescent dye and transfer it to MDA-MB-231 recipient cells (Figure 4).

We showed the accumulation of the fragments of mutant DNA or RNA transcripts in the exosomes of the cancer cells. Based on our data and data from other researchers showing the exosomes’ ability to incorporate into the recipient cells, we can propose the possible involvement of the exosomal tumour DNA in the recipient cell rearrangement. The latter one agrees with the earlier observations showing the exosomes’ capability to transfer the fragments of tumour DNA to recipient cells, including the microenvironment cells, and to induce in those cancer-associated changes [12,13,14,15]. We did not find a detectable amount of the mutant DNA in the exosome-treated recipient cells—probably because of the extremely low amount of the incorporated DNA. A similar problem was pointed out by other authors who noted the low efficiency of the detection of heterologous exosomal DNA in the recipient cells [13]. 

The origin of the exosomal DNA is still unclear; it may be the micronuclei resulting from chromosome aberrations during mitosis [16], cytoplasmic fragments of the damaged DNA [17], and DNA transposons or cDNA reversed from retrotransposons [18]. Another important problem is whether the possibility of the integration of the heterologous exosomal DNA into the genomic DNA of the recipient cells exists. Single publications demonstrate the transfer of exosomal DNA into the recipient cells accompanied by stable integration of the DNA and transmission to daughter cells after some passaging [13,19]. In general, the presence of the mutant DNA in the exosomes does not seem to be sufficient to induce the malignant transformation of the recipient cells, and the combination of tumour exosomes with the additional cancer-initiating agents is required. 

## 3. Materials and Methods

The human breast cancer cell lines MCF7 and MDA-MB-231 were purchased from ATCC. The cells were authenticated by morphology and STR profiling provided by Gordiz (http://gordiz.ru/, accessed on 12 August 2020). The cells were cultured in standard DMEM medium (Gibco) supplemented with 10% foetal bovine serum (FBS) (HyClone) at 37 °C and 5% CO_2_. Exosomes were prepared from the MCF7 conditioned medium by the differential ultracentrifugation and were characterized as described in our recent paper [11]. Transmission Electron Microscopy (TEM) was used to visualize the exosome samples. They were deposited onto the carbon-coated grids (Ted Pella, Redding, USA) pre-treated using a K100X glow discharge unit (Quorum Technologies Ltd, Lewes, Great Britain) and stained with 1% uranyl acetate (Electron Microscopy Sciences, Hatfield, UK). Imaging was carried out using a JEM-1011 transmission electron microscope (JEOL Ltd., Tokyo, Japan) at 80 kV. 

To reduce external DNA contamination, before DNA extraction, exosomes were treated with DNase I, and exosomal DNA was extracted by phenol/chloroform mixture; total exosomal RNA was extracted in Trisol.

For cell treatments, MCF7 exosomes were added to the MDA-MB-231 cells for 3 days at the final concentration of 1.7 μg/mL of exosomal protein or (5.5 ± 0.3) × 10^9^ vesicles/mL; then the cells were washed with PBS, and cytoplasmic DNA and total RNA were extracted and subjected for sequencing. For exosome labelling, the MCF7 vesicles were incubated with fluorescent dye CellTracker™ Red CMPTX Dye (Thermo Fisher Scientific, Waltham, MA, USA) and washed in PBS by the ultracentrifugation for 2 h at 100,000× *g*. Precipitates were dissolved in PBS and incubated with MDA-MB-231 cells for 1 h before fluorescent microscopy analysis.

cDNA conversion: cDNA synthesis was carried out with 1 µg total RNA by using the MiScript Reverse Transcription Kit (Qiagen, Hilden, Germany) according to the manufacturer’s protocol.

Sanger sequencing: The fragments with *PIK3CA* (p.Glu545Lys) mutation were amplified using specific primers from nuclear and exosomal DNA and cDNA (Table 2).

The direct sequencing of individual PCR products from primers that flank areas of specific mutations was performed on the automatic genetic analyser 3500 (Thermo Fisher Scientific, Waltham, MA, USA) according to the manufacturer’s protocols.

Real-time PCR. The *PIK3CA* mutation c.1633G > A (p.E545K) was detected by allele-specific real-time PCR with TaqMan probes using TaqMan^®^ Mutation Detection Assay (Hs00000824_mu/Hs00001025_rf assay, recommended reagents, consumables, and protocols; Thermo Fisher Scientific, Waltham, MA, USA) and DT-Prime thermocycler (DNA-Technology, Moscow, Russia). The mutant and normal (reference) alleles of the same site of the *PIK3CA* gene were amplificated; DNA from MDA-MB-231 cells (originally contained a wild type of *PIK3CA*) was used as a negative control.

## 4. Conclusions

The results demonstrate the exosome-mediated secretion of the fragments of mutant DNA and mRNA by the cancer cells and demonstrate the exosomes’ ability to incorporate into the heterologous cells. We propose that further investigations will delineate the role of the exosome-transferred mutant DNA in the tumour progression as well as in the formation of the cancer-associated phenotype of the cells of the tumour microenvironment.

## Figures and Tables

**Table 1:** Allele-specific real-time PCR of the DNA samples of exosome-treated cells.

Sample	Reference Allele				Mutant Allele				Delta Ct	Delta Ct Cut-off	Mutation
	Ct (1)	Ct (2)	Ct (3)	Average Ct	Ct (1)	Ct (2)	Ct (3)	Average Ct			
MCF7 cells	15.1	15.3	15.7	15.4	19.1	19.2	19.2	19.2	3.8	11.5	Yes
MDA-MB-231 cells	17.4	17.4	17.4	17.4	29.7	30.2	30.5	30.1	12.7		No
MDA-MB-231 cells + exoMCF7	15.9	15.9	16	15.9	26.1	26.2	26.3	26.2	10.3		Yes
RT-PCR negative control	-	-	-	-	-	-	-	-	-		No

**Table 2:** Sequence primers *PIK3CA* detection.

	Sequence Primers *PIK3CA* (p.Glu545Lys)	Fragment Length	T
DNA	F: gggaaaaatatgacaaagaaagc R: ctgagatcagccaaattcagtt	250 bp	60
c-DNA	F1: ccacgcaggactgagtaaca R1: ggccaatcttttaccaagca	246 bp	60

## Data Availability

Datas are available from the authors.

## Sample Availability

Samples are available from the authors.
